# Perceptions of Fatigue and Safety Climate Pertaining to Residency Duty-Hour Restrictions

**DOI:** 10.7759/cureus.28929

**Published:** 2022-09-08

**Authors:** Michele M Carr, Jonathan Friedel, Daniel O'Brien, Anne M Foreman, Oliver Wirth

**Affiliations:** 1 Otolaryngology, Jacobs School of Medicine and Biomedical Sciences at the University of Buffalo, Buffalo, USA; 2 Psychology, Georgia Southern University, Statesboro, USA; 3 Otolaryngology, West Virginia University, Morgantown, USA; 4 Bioanalytics Branch, Health Effects Laboratory Division, National Institute for Occupational Safety and Health, Centers for Disease Control and Prevention, Morgantown, USA

**Keywords:** sleep, duty hours, safety climate, physician fatigue, resident education

## Abstract

Introduction: The Accreditation Council for Graduate Medical Education (ACGME), which sets the standards for residency training, instituted work-hour restrictions in 2003. Our purpose was to assess residents’ perceptions of fatigue and local safety climate specific to these duty-hour restrictions.

Methods: All residents (N=433) at one university were emailed a link to a survey in 2019. The survey included demographic details, on-call descriptors, an 18-point climate survey (CS), and the 33-point Chalder Fatigue Questionnaire (CFQ). The CS was adapted from a commonly used safety climate scale and intended to measure the respondent’s perceptions of their program’s attitudes and practices around resident duty-hour compliance. A Pearson correlational analysis was used to determine if there were associations between the variables.

Results: Mean CS score was 12.89 (95% confidence interval, CI 12.32-13.46, N=164, 48.5%). Respondents were most likely to disagree with “Residents are told when they are at risk of working beyond ACGME duty-hour restrictions,” where 57 (34.7%) disagreed or strongly disagreed. Mean CFQ score was 16.02 (95% CI 14.87-17.17, N=113, 26.1%). As the CS score improved, CFQ scores decreased indicating an inverse relationship between duty-hour climate and fatigue (r=-0.328, p<0.05). Having a protected post-call day off, and having either the Program Director, Chief Resident, or Senior Resident decide that a resident takes a post-call day off were all associated with higher CS scores.

Conclusion: We found that the CS had good internal consistency and evidence of construct validity. An inverse relationship between CS score and fatigue suggests that the level of fatigue is higher among residents in programs where residents perceived that ACGME duty-hour compliance was less important.

## Introduction

There is clear evidence that fatigue affects cognitive and motor performance [1]. Fatigue in medical training came into the public eye after the 1984 death of 18-year-old Libby Zion within 8 h of her emergency admission to a New York teaching hospital, where she had been cared for by junior residents. A 1986 grand jury investigation decided that the death was related to 36-h shifts worked by the residents involved in her care and to inadequate supervision by the attending physicians [2]. Their recommendations were incorporated into the New York State Health Code in 1989, making New York the first state to limit resident work hours to 80 per week [2]. However, by 2002 compliance was evident in only 60% of teaching hospitals in that state [2]. The Accreditation Council for Graduate Medical Education (ACGME), which sets the standards for residency training, instituted work-hour restrictions in 2003 that were redefined in 2011 [2]. Despite the costs of implementing these restrictions, there is little evidence that it is improving patient safety as intended [3].

Evidence suggests that non-compliance with duty-hour restrictions remains a major concern. For example, one study found no difference in work hours or sleep hours in residents after scheduling changes to accommodate ACGME duty-hour standards were made [4]. In the occupational safety and health literature, compliance with safety-related policies and practices in an organization has been linked to the prevailing safety culture. Safety culture refers to a set of norms, values, perceptions, and beliefs that govern behavior and ultimately safety outcomes [5]. The similar concept of safety climate [6] concerns employee perceptions of the safety culture in an organization [7]. Safety climate has been shown to be an important predictor of adherence to safe work practices [8]. Safety climate in medicine, specifically pertaining to compliance with ACGME policies, has not been investigated. In this study our goal was to assess fatigue and a novel measure of safety-related climate that pertains specifically to ACGME duty-hour restrictions in residency.

## Materials and methods

This study was approved by the West Virginia University Institutional Review Board. All university Graduate Medical Education (GME) residents (N=433) were contacted by direct email and sent a link to the survey. Addresses were provided by the institution’s Office of Graduate Medical Education. Incentive for participating was a $5 gift card given electronically several weeks after survey completion. A maximum of four mailings were sent to non-respondents between February and June 2019, at approximately one-month intervals.

 Study data were collected and managed using Research Electronic Data Capture (REDCap) electronic data capture tools hosted at West Virginia University [9-10]. REDCap is a secure, web-based platform designed to support data capture for research studies.

 The results reported here were part of a larger survey that included demographic information, the Chalder Fatigue Questionnaire (CFQ) [11], a modification of a common six-item safety climate scale for measuring workplace safety climate [12], and a discrete choice experiment that will be the focus of a future study and thus is not reported here. The CFQ has been applied in clinical and community populations by many research teams; scores range from 0 to 33 in total [11]. It includes questions about feeling tired or drowsy, muscle weakness, having issues with speaking, having difficulty concentrating, having difficulty starting things, and memory concerns [11, 13]. The CFQ has been shown to have a Cronbach’s α of 0.88-0.92, with good discriminant ability in higher scores; a score of 29 predicts chronic fatigue with 95% accuracy [11, 13]. 

The original safety climate scale has been administered in hospital environments with nurses, technologists, and physicians with measures of internal consistency ranging from 0.71 to 0.85 [12]. Our modified version (hereafter, climate scale or CS) aimed to measure respondents’ perceptions of workplace safety culture with regard to adherence to ACGME duty-hour restrictions and preservation of resident health and safety, in general. As with the original safety climate scale, the CS used herein assessed safety-related climate across four main dimensions: behavioral norms, supervisory performance feedback, management commitment to safety, and worker involvement. These dimensions of safety climate, in various forms, are common to several other safety climate surveys (CSs) used in healthcare [14]. The modifications were devised by a team of occupational health scientists and academic surgeons. The original and modified surveys appear in Table 1. Total scores range from 0 to 18.

**Table 1:** Questions on the SCS scales. Comparison of questions on the SCS scales. The original scale was modified to reflect the safety aspects of resident fatigue. SCS, safety climate survey; ACGME, Accreditation Council for Graduate Medical Education

	Original six-item safety climate survey questions	Modified six-item sleep safety climate survey questions	Safety climate dimension [12]
1	New employees learn quickly that they are expected to follow good health and safety practices.	New residents in our program quickly learn that they are expected to adhere to the ACGME duty-hour restrictions.	Behavioral norms
2	Employees are told when they do not follow good health and safety practices.	Residents are told when they are at risk of working beyond ACGME duty-hour restrictions.	Supervisory performance feedback
3	Workers and management work together to ensure the safest possible conditions.	In my program, residents and their supervisors work together to ensure that residents can follow ACGME duty-hour rules.	Management commitment
4	There are no major shortcuts taken when worker health and safety are at stake.	There are no significant compromises or shortcuts taken when residents' health and safety is at stake.	Management commitment
5	The health and safety of workers is a high priority with management where I work.	The health and safety of residents is a big priority with their Program Director.	Management commitment
6	I feel free to report safety problems where I work.	I feel free to report deviations from ACGME duty-hour restrictions.	Worker involvement

Statistical analyses were completed using R [15] with the addition of the “Tidyverse” packages [16]. Pearson’s r values were calculated with the “corrr” package [17] and Cronbach’s α was calculated with the “psych” package [18]. We chose not to conduct a correction to account for a false discovery rate [19] because of the relatively small number of correlations that were calculated.

## Results

The total response rate for the survey was 42.7% (n = 181), defined as respondents who initiated the survey and completed at least one question. There was a moderate degree of attrition of respondents between the CS questions and the final question of the survey. For that reason, we report the demographics only for respondents who completed the CS (90.6% of respondents who initiated the survey, n = 164). Table 2 shows the demographic information for the 164 respondents who completed the CS. Thirty-one (18.9%) were in internal medicine, 21 (12.8%) were in family medicine, 14 (8.5%) were in pediatrics, 11 (6.7%) were in surgery, 24 (14.6%) were from six other programs, and the remaining 63 (38.4%) declined to identify their residency program.

**Table 2:** Demographic characteristics of the survey participants (n=164). ^*^Note: respondents could select multiple options for this question.

	N (%)
Postgraduate year	
1	53 (32.2)
2	43 (26.2)
3	41 (25.0)
4	20 (12.2)
5	4 (2.4)
6	2 (1.2)
Not stated	1 (0.6)
Age	
24 years or younger	1 (0.6)
25-29 years	83 (50.6)
30-35 years	65 (39.6)
36 years or older	11 (6.7)
Not stated	4 (2.4)
Gender	
Male	87 (53.0)
Female	70 (42.7)
Not stated	7 (4.2)
Children	
Have children in their home	33 (20.1)
On call description	
In-house call	44 (26.8)
Night float	44 (26.8)
At-home call	13 (7.9)
Other or not stated	63 (38.4)
Taking primary call this academic year	
Yes	76 (46.3)
No	32 (19.5)
Not stated	56 (34.1)
Has protected post-call days off	
Yes	87 (53.0)
No	26 (15.9)
Who decides if you have a post-call day off?^*^	
Self	26 (15.9)
Senior Resident	17 (10.4)
Chief Resident	57 (34.8)
Attending Faculty	10 (6.1)
Program Director	43 (26.2)
Department Chair	8 (4.9)
Other	4 (2.4)
Unsure	32 (19.5)
Program	
Internal Medicine	31 (18.9)
Family Medicine	21 (12.8)
Pediatrics	14 (8.5)
Surgery	11 (6.7)
Orthopedics	8 (4.9)
Ophthalmology	5 (3)
Radiology	4 (2.4)
Obstetrics-Gynecology	3 (1.8)
Otolaryngology	2 (1.2)
Pathology	2 (1.2)
Other	63 (38.4)

**Figure 1:**
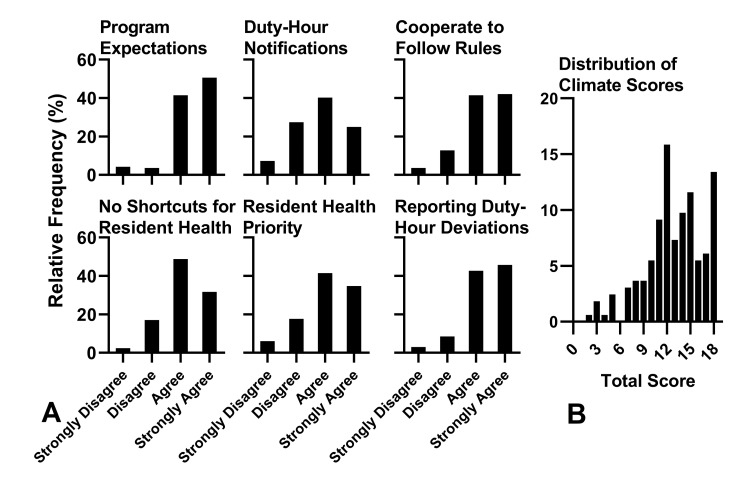
Distribution of scores for individual questions in the CS. N=164. A: Distribution of scores for individual questions in the CS. N=164. B: Distribution of total CS scores. N=164. CS, climate survey

 Figure 1 shows the results of the CS. Cronbach’s α for the survey was 0.85, indicating a high degree of internal consistency. The mean score was 12.89 (95% CI: 12.32-13.46). Respondents were most likely to disagree with “Residents are told when they are at risk of working beyond ACGME duty-hour restrictions,” where 57 (34.7%) indicated that they disagreed or strongly disagreed. Thirty-nine (23.8%) disagreed or strongly disagreed with “I feel free to report deviations from ACGME duty-hour restrictions,” and 32 (19.5%) disagreed or strongly disagreed with “There are no significant compromises or shortcuts taken when residents' health and safety is at stake.”

**Figure 2:**
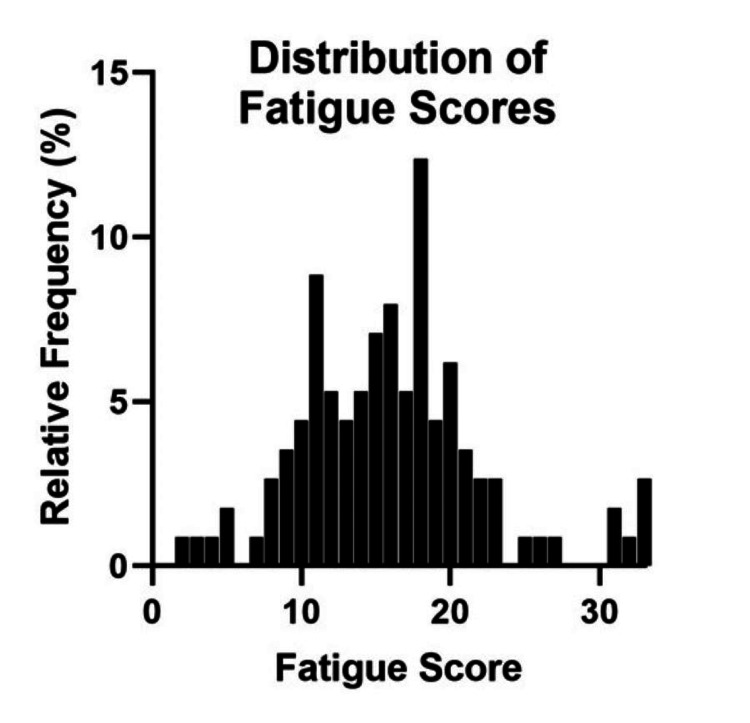
Distribution of total CFQ scores. Higher scores indicate greater fatigue. N=113. CFQ, Chalder Fatigue Questionnaire

 One hundred thirteen respondents completed the entire survey (61.4% of respondents who initiated the survey). Cronbach’s α was 0.93. Mean score was 16.02 (95% CI 14.87-17.17), which is between “No worse than usual” (total score of 11 for all responses) and “Worse than usual” (total score of 22). Results are shown in Figure 2.

 There were several significant correlations worth noting that are included in Table 3 (significance level was set to 5%). As the CS score increased, indicating a better perceived safety climate, total scores on the CFQ and the CFQ memory question decreased, indicating less fatigue and better memory, respectively. Having a protected post-call day off as well as having either the Program Director, Chief Resident, or Senior Resident deciding when to take a post-call day off were all associated with higher CS scores, indicating better safety climates. Having the Department Chair decide if a resident takes a post-call day off was associated with lower CFQ scores, indicating less fatigue. Respondents indicating that they were unsure who decides if a resident takes a post-call day off had lower CS scores.

**Table 3:** Notable correlations between CS score, CFQ score, and demographic characteristics. p < 0.05 ^*^These correlations should be interpreted cautiously because of the relatively small cell size of people responding “yes” CS, climate survey; CFQ, Chalder Fatigue Questionnaire

Comparator	Scale	r	Interpretation
CS score	CFQ score	-0.328	CS score decreases as CFQ score increases
Department Chair decides post-call day off	CFQ score	-0.186^*^	Dept chair deciding post-call day off associated with decreasing CFQ score
Has protected post-call day off	CS score	0.262	Having a protected post-call day off is associated with increased CS score
Senior resident decides if someone has a post-call day off	CS score	0.265^*^	Having a senior resident decide if someone has a post-call day off is associated with increased CS score
Chief resident decides if someone has a post-call day off	CS score	0.269	Having a chief resident decide if someone has a post-call day off is associated with increased CS score
Program Director decides if someone has a post-call day off	CS score	0.244	Having a program director decide if someone has a post-call day off is associated with increased CS score
Respondent is unsure who decides if someone has a post-call day off	CS score	-0.227	Respondents who don’t know who decides if someone has a post-call day off have lower CS scores

## Discussion

This study provides evidence of associations among self-reported fatigue and local attitudes, perceptions, and practices pertaining to ACGME duty-hour restrictions in one university’s residency programs. Most respondents had positive perceptions of the safety-related climate around duty-hour policies and practices with respect to the dimension of behavioral norms. This indicates that respondents were aware of the duty-hour restrictions and the program’s expectations for compliance. Although the total climate scores were mostly positive, small, but appreciable, percentages of respondents had negative perceptions of safety-related climate around the dimensions of management commitment (19.5%), and supervisory feedback (34.7%), and worker involvement (23.8%), indicating areas for improvement. The measure of safety-related climate, which was adapted to focus specifically on resident duty-hour compliance for this study, was partially validated. We found both high internal consistency and a measure of construct validity as scores on the CS were inversely correlated with fatigue scores. This demonstrated the potential for the scale’s construct validity because the duty-hour restrictions aim to reduce excessive fatigue in residents, and residents who perceived better institutional compliance with the duty-hour restrictions reported less fatigue. Higher CS scores also were positively correlated with residents' reports that their program had protected post-call days off, and with residents reporting that persons with authority within the program decide when a resident takes a post-call day off. Putting enforcement decisions about compliance with duty-hour restrictions into the hands of authority figures suggests that a residency program’s leadership values a commitment to health and safety, a finding that also supports the construct validity of the CS.

 There is a paucity of evidence in the literature that ACGME duty-hour policies are associated with improved resident wellness, including fatigue, or that resident fatigue is clearly associated with patient safety or medical errors. In the last 20 years, the ACGME has put a lot of attention toward controlling resident duty hours in the expectation that fewer hours would improve fatigue and thereby improve patient safety [2, 20]. Numerous studies have subsequently attempted to evaluate outcomes of interventions that reduce work hours such as reducing shift length, using a night float system, or having protected sleep time. The most recent systematic review on this topic examined 27 articles for effect of these strategies on patient care, resident wellness, and resident education [3]. With respect to the impact on patient care, 4 of 10 studies showed a favorable impact, 5 showed no change, and 1 showed an unfavorable impact. Fourteen studies looked at the impact of interventions to reduce work hours on resident education; nine studies found an unfavorable impact, three found no change, and only one found a favorable impact. In a review of duty-hour restrictions in orthopedic residencies, the effects of work hour restrictions on case volume were equivocal, as were effects on patient care [21]. In a study conducted during the 2014-2015 academic year, 117 general surgery residency programs were cluster randomized into either a group following ACGME duty-hour rules or a group using a flexible policy that waived rules on maximum shift lengths and time off between shifts [22]. This study reported no significant differences between the groups with respect to complications, as measured by ACS-NSQIP, over the course of the study [22]. However, this method of accruing data about complications does not measure medication errors and near misses.

At this point, there is no clear evidence that following ACGME mandated duty-hour restrictions has any positive effect on patient care. There are several reasons why this may be true [23]. It has been postulated that duty hours are a minor component of resident fatigue, and resident fatigue is in turn a minor determinant of harmful medical errors [23]. It has also been suggested that there are adverse effects of reducing duty hours, such as less time interfacing with teaching faculty and reduced continuity of care [23]. Landrigan et al.’s study, described in the introduction to this article [4], leads us to suspect that duty-hour limits were not being observed in that program, because work hours and sleep hours did not change after institution of restrictions. This has also been reported in internal medicine residencies via a similarly designed study [24].

Limitations

In our study we are focusing on duty-hour restrictions, but this is only one factor associated with a program’s safety culture. Measuring other programmatic and administrative aspects of residency programs, such as resident and faculty wellness and burnout and patient safety report rates may give more information about general safety climate. Additionally, we did not measure the actual number of duty hours each respondent worked in a typical week. The actual number of hours worked by a resident is difficult to ascertain because individuals may be knowingly violating duty-hour violations for a variety of reasons. Asking a respondent to self-identify as violating duty-hour restrictions is sensitive and would likely increase non-response and attrition of respondents. This may be an area for future study.

 This study was undertaken at a single university center, and the response rate was only 42.7%, both of which limit generalizability. Furthermore, the samples from individual residency programs were small, so we were not able to reliably compare measures (e.g., CFQ, CS) across programs within the institution. A large percentage of respondents (38%) chose not to identify their program, which also prevents us from making conclusions about specific programs. Lastly, fatigue is self-reported and reflects self-perception, so scores may not be representative of actual fatigue.

## Conclusions

We have described and begun to validate a safety-related CS that assesses attitudes and perceptions of ACGME duty-hour policies and practices. We found a correlation between increased fatigue and lowered climate scores, suggesting that residents are more fatigued in environments where they perceive that ACGME duty-hour compliance is less important. This has not been described previously and is an area for ongoing attention.
